# Haptic and Palpation Sensing for Robotic Surgery: Engineering Perspectives on Design and Integration

**DOI:** 10.3390/s26041126

**Published:** 2026-02-10

**Authors:** Michael H. Friebe

**Affiliations:** 1 Faculty of Computer Science, Heathtech Innovation Lab, AGH University of Krakow, 30-059 Krakow, Poland; friebe@agh.edu.pl; 2 Faculty of Medicine, Otto-von-Guericke University, 39106 Magdeburg, Germany; 3 5P Future of Health GmbH, 44801 Bochum, Germany

**Keywords:** Robotic-assisted surgery, palpation sensing, haptic feedback, tactile and force sensors, vibroacoustic sensing, multimodal sensor fusion, artificial intelligence, surgical autonomy

## Abstract

**Highlights:**

Robotic-assisted surgery (RAS) lacks clinically integrated palpation and haptic feedback. Emerging force, tactile, vibroacoustic and audio sensors enable quantifiable tissue characterisation. Sensor performance can exceed human tactile sensitivity but remains difficult to integrate clinically. Multimodal sensor fusion with artificial intelligence and imaging is essential to compensate for variability and noise. Palpation-enabled RAS supports improved safety, training efficiency, and progression toward autonomous operation.

**What are the main findings?**

- This perspective identifies the absence of palpation and haptic sensing as a key limitation of current robotic-assisted surgical systems. While multiple sensor technologies are capable of quantitatively capturing tissue mechanical properties, their clinical adoption is restricted by challenges in miniaturisation, sterilisation, robustness, and system integration. The analysis shows that sensor sensitivity already exceeds human tactile thresholds, enabling objective tissue assessment and personalised feedback. However, the lack of standardised benchmarks, clinically grounded validation protocols, and seamless integration into existing RAS platforms remains a major barrier.

**What are the implications of the main findings?**

- Integrating robust, high-fidelity palpation and haptic feedback into RAS could shorten learning curves, reduce tissue damage, and improve precision and surgeon confidence, particularly in complex minimally invasive procedures. Combining compact, affordable sensing hardware with AI, AR/VR, and imaging is a prerequisite for semi-autonomous and, eventually, autonomous robotic surgery and will require standards, validation studies, and cost-effective designs to achieve broad global adoption.

**Abstract:**

Robotic-assisted surgery (RAS) provides enhanced dexterity and visualisation but remains constrained by the absence of clinically meaningful palpation and haptic feedback. This perspective examines palpation sensing in RAS from an engineering and system-integration standpoint, identifying the lack of tactile information as a major contributor to increased cognitive load, prolonged training, and risk of tissue injury. Recent advances in force, tactile, vibroacoustic, audio, and optical sensor technologies enable quantitative assessment of tissue mechanical properties and often exceed human tactile sensitivity. However, clinical translation is limited by challenges in sensor miniaturisation, sterilisation, robustness and integration and the absence of standardised evaluation metrics. The integration of artificial intelligence and multimodal sensor fusion with intra-operative imaging and augmented visualisation is highlighted as a key strategy to compensate for sensor limitations and biological variability. Dedicated robotic palpation devices and wireless or magnetically coupled probes are discussed as promising transitional solutions. Overall, the restoration of palpation sensing is presented as a prerequisite for improving safety and efficiency and enabling higher levels of autonomy in future RAS platforms.

## 1. Introduction—Need for Palpation in Robotic Surgery

There is a growing need to develop and integrate a clinically meaningful palpation sense into next-generation robotic surgical systems. This perspective presents the topic from an engineering, system-design, and innovation development perspective.

### 1.1. Definition of Key Terms

Robotic-assisted surgery (RAS) is a form of minimally invasive surgery in which the surgeon performs an operation by controlling computerised robotic instruments, typically via a console that provides magnified three-dimensional in situ visualisation and precise, tremor-filtered instrument movements, with the goal of enhancing dexterity, accuracy, and access in complex procedures, while the surgeon maintains full control at all times [1]. The advantages of robotic surgery are mainly the minimisation of the surgical invasiveness through the use of smaller incisions, resulting in reduced blood loss, and accelerated patient recovery [2].

Palpation in robotic-assisted surgery refers to the deliberate probing or pressing of tissue with an instrument to assess mechanical and structural properties like stiffness and texture and the presence of hidden structures. Tactile, on the other hand, refers to surface touch information (e.g., local pressure, texture, small bumps) captured by distributed pressure or touch sensors and conveyed back to the surgeon or control system.

Haptic feedback denotes the overall sense of touch in RAS, combining tactile and force/kinesthetic cues and delivering them via feedback (e.g., vibration, force reflection) to recreate touch at the surgeon console.

Kinesthetic feedback on the other hand relates specifically to the perception of gross forces, torques, and motion of the surgeon’s hands or robot joints (e.g., resistance when pulling or pushing), often provided through force feedback at the master manipulators [3,4].

Palpation and haptic feedback are critical not only for enhancing surgical performance and improving clinical outcomes but also for enabling data-driven and predictive models that support intra-operative decision-making. These capabilities will be essential for advancing toward semi-autonomous and fully autonomous RAS platforms [5,6].

Currently, most RAS systems offer limited autonomy and typically lack palpation or haptic feedback, relying predominantly on visual input from integrated camera systems. This limitation necessitates extensive preoperative training, thereby reducing the pool of qualified operators and limiting procedural scalability. The development of advanced educational tools for minimally invasive surgery and therapy (MIS/MIT) and RAS is, therefore, increasingly important, as is the integration of sensor technologies and artificial intelligence (AI)-based tools that reduce operator dependency and variability.

***“A development goal for RAS systems must be to eventually provide high-quality, accessible, forward-looking (outcome predictive), intelligent and affordable surgical solutions anywhere in the world (democratising access). This is only possible with scalable, more autonomous robotic surgery systems built at scale that are not fully dependent on a human clinical expert.”***

The intention of this perspective is to provide an engineering-focused overview of emerging technologies and research directions with a high potential for translation and integration into future RAS systems. Emphasis is placed on approaches that reduce system complexity, development costs, and operational burden while maintaining clinical relevance and technical feasibility.

### 1.2. Goal of This Engineering Perspective

This perspective argues that the restoration of palpation and haptic sensing is a foundational prerequisite for safe, scalable, and more autonomous robotic-assisted surgery and that this will require integrated, affordable, and robust sensor technologies tightly coupled with AI and imaging. Palpation and haptic feedback are not optional refinements but essential capabilities that current RAS systems largely lack, thereby constraining tissue characterisation, limiting autonomy, and impeding global scalability despite excellent vision and kinematics.

Future surgical robots should be defined as intelligent, sensing-rich systems that combine force, tactile, vibroacoustic, audio, visual, and other sensing modalities with AI and intra-operative imaging to approximate, or ultimately exceed, the diagnostic value of human touch in MIS/RAS.

The following core claims are defended:

1. The absence of tactile and palpation feedback in current RAS is a major bottleneck, as it increases training time and cognitive load, raises the risk of tissue injury from excessive forces, and limits progression toward higher levels of autonomy and cost-effective global deployment.
2. Emerging sensor modalities, including force and tactile sensors at the tip/shaft; vibroacoustic and audio sensing; soft-robotic and tactile elements; sensorised tools; and wireless or magnetically coupled palpation probes, can capture clinically relevant tissue mechanics. However, these approaches remain fragmented, are challenging to miniaturise and sterilise, and are rarely integrated into commercial RAS platforms.
3. Quantitative palpation is technically feasible, and sensor performance today already exceeds human tactile sensitivity, enabling objective tissue characterisation and individualised haptic mappings rather than purely subjective perception.
4. Robust, compact, and affordable palpation and haptic solutions, when combined with AI, augmented/virtual reality (AR/VR), and intra-operative imaging, represent key levers to shorten learning curves, reduce tissue damage, improve decision-making, and progress from purely tele-operated assistance toward semi-autonomous and, eventually, autonomous RAS.
5. Finally, sensor noise, drift, tissue and interoperator variability, and integration constraints, such as size, sterilisation, and biocompatibility, currently prevent reliable and reproducible “touch” in RAS. Future work must, therefore, prioritise advanced compensation algorithms, standardised metrics for sensor performance evaluation, and clinically grounded validation protocols.

## 2. Status Quo and Definitions

Skin palpation (from Latin *palpatio*—to touch) is a fundamental clinical examination technique performed after visual inspection to assess tissues, organs, bones, joints, and soft tissues. Through touching, pressing, and stroking, clinicians gather primarily qualitative—and to a limited extent quantitative—information about temperature changes and gradients, moisture, tenderness, pulsatility, swelling, and the presence of masses, deformities, or abnormal compliance.

In orthopaedic surgery, for example, palpation remains a crucial diagnostic tool. It helps clinicians to localise sources of pain or tenderness, identify inflammation or infection through temperature assessment, evaluate vascular status via pulse detection, and detect structural abnormalities or characterise soft tissue and bony masses.

While open surgeries rely on direct human palpation, minimally invasive surgery (MIS) and minimally invasive techniques (MIT) offer numerous advantages, including reduced postoperative pain, shorter hospital stays, and faster recovery. However, a key drawback—particularly in robotic-assisted procedures—is the loss of tactile and kinesthetic feedback due to the indirect interaction between instruments and patient tissue (see Table 1 for additional limitations). This sensory gap can lead to excessive or poorly modulated force application, increasing the risk of tissue damage and procedural complications [7,8].

***“The absence of haptic feedback is particularly pronounced in RAS where the physical separation between the surgeon and the patient exacerbates the lack of direct touch sensation.”***

To compensate for the lack of tactile feedback, surgeons rely heavily on visual cues from endoscopic video, occasionally supplemented by external or intra-operative imaging. They also invest substantial time in training and experience, which limits the pool of surgeons capable of performing advanced procedures. While these strategies can partially mitigate performance degradation, they may prolong procedure times and increase complication rates compared to open surgery, where tactile feedback is inherently available. Consequently, there is a critical need for advanced haptic feedback systems that enhance a surgeon’s perception of tissue mechanics by integrating visual and tactile information, thereby optimising motor control, decision-making, and overall surgical performance [9].

### 2.1. Robotic Surgery Systems (RAS)

RAS systems enhance surgical precision, dexterity, and visualisation, enabling smaller incisions, reduced blood loss, and faster patient recovery compared to traditional open or even standard minimally invasive surgery [10]. Nevertheless, RAS systems continue to face significant limitations in tissue characterisation and palpation.

In conventional surgery, surgeons rely on both kinesthetic (forces and torque) and tactile (surface texture, compliance, and vibration) feedback to assess tissue properties. Robotic systems, while improving visual capabilities, largely eliminate these critical sensory cues [11]. Without palpation, distinguishing between healthy and pathological tissue or detecting subtle changes in consistency indicative of disease becomes substantially more challenging. Although advanced imaging and visualisation tools can assist intra-operative assessment, they do not fully replicate the nuanced information provided by direct touch [12].

RAS platforms typically feature articulated instruments that replicate the surgeon’s hand motions while providing tremor-free precision and enhanced visual access. These features have driven adoption across a wide range of surgical specialties, including:

**General Surgery**: cholecystectomy, hernia repair, appendectomy;

**Gynaecology**: hysterectomy, myomectomy, ovarian cystectomy;

**Urology**: prostatectomy;

**Cardiac Surgery**: mitral valve repair, atrial fibrillation ablation;

**Orthopaedics**: total knee and hip arthroplasty;

**Spinal Surgery:** spinal fusion.

Currently, most robotic surgical systems are neither autonomous nor semi-autonomous. Instead, they are best characterised as tele-operated, robot-assisted systems, with the surgeon retaining full control over all aspects of the procedure. A recent systematic review using the *Levels of Autonomy in Surgical Robotics (LASR)* scale found [13] (see Table 2):

**Level 1 (Robot Assistance)**: Most FDA-cleared systems fall into this category, offering cognitive and physical assistance without independent decision-making.

**Level 2 (Task Autonomy)**: A few systems can perform specific, preprogrammed tasks (e.g., suturing or cutting) when initiated by the surgeon but cannot plan or act independently.

**Level 3 (Conditional Autonomy)**: Some advanced platforms can generate patient-specific plans or adapt intra-operatively yet still require surgeon oversight for critical steps.

No robotic systems currently in clinical use operate at **Level 4 (High Autonomy)** or **Level 5 (Full Autonomy)**, in which the robot would independently plan and execute procedures without human intervention.

Some surgical systems are beginning to incorporate AI and machine learning for specific tasks, such as patient-specific implant positioning or 3D-augmented reality overlays. However, these capabilities currently assist rather than replace the surgeon.

Achieving higher levels of autonomy in robotic surgery will require richer sensory input from the surgical site, real-time imaging, and advanced situational analysis powered by AI models trained on complete procedures and capable of predicting outcomes based on evolving intra-operative data.

Without increased autonomy, the scalability of robotic-assisted surgery (RAS) remains limited, as the presence of a trained human operator is still required. True scalability and remote operation will only become feasible when systems can function independently of the surgeon’s physical presence.

***“Palpation sensation is not merely advantageous but essential for enabling higher autonomy in robotic surgery. If achieved, it could bridge the gap between human tactile expertise and robotic precision, promoting safer and more accurate interventions.”***

Integrating additional sensors with AI-based analysis can enhance tissue discrimination and facilitate pathology detection by identifying variations in stiffness, elasticity, and compliance. For example, autonomous systems equipped with force sensors and deep learning can estimate the depth of hard inclusions (e.g., tumours) within soft tissue, effectively mimicking human palpation [14,15].

Multimodal feedback systems—such as vibrotactile or pneumatic interfaces—have been shown to significantly improve localisation of tumours and vessels during robotic palpation. Recent haptic-enabled robotic systems demonstrate substantial reductions in tissue loading, with up to 43% lower applied force in commercial feedback trials (da Vinci 5, Intuitive Surgical, Sunnyvale, CA, USA) and approximately 40–55% reductions reported in experimental haptic-feedback studies. In addition, integrated optoelectronic force/torque sensors can achieve approximately 95% measurement accuracy for surgical tool loads [3,16,17].

Machine learning models trained on tactile and force data enable robots to adapt to dynamic surgical environments by compensating for tissue deformation during manipulation. Surgeons report increased confidence and control when using systems equipped with haptic feedback, indicating that similar capabilities will be critical for semi-autonomous systems to achieve widespread clinical acceptance [18,19].

***“Challenges persist in standardising and amplifying force feedback in robotic platforms.”***

Research efforts are ongoing to develop systems that accurately replicate the sensory response of the human nervous system without introducing latency or communication delays—factors that are critical for surgical safety.

The “feel” of tissue in robotic-assisted surgery can be quantified by measuring mechanical properties at the tool–tissue interface, including force–displacement behaviour, stiffness or compliance, and viscoelastic response. These measurements are obtained through instrumented palpation or grasping and relating these values to perception scales derived from surgeon studies. The robotic palpation devices are correlated with perception scales derived from surgeon studies. Robotic palpation devices and tactile sensors generate force–deformation curves and estimate in situ elastic modulus or effective spring constants, enabling numerical comparison of “soft”, “firm”, and “hard” tissue regions and supporting pathology detection. Because surgeons differ in their thresholds and just-noticeable differences for stiffness perception, haptic feedback should be individually calibrated. In most cases, sensor sensitivity exceeds human tactile perception, making such personalisation technically feasible [5,20].

Table 3 summarises the current status of efforts to simulate or replicate the tactile sensations associated with palpation, comparing human capabilities with the implementation of corresponding robotic technologies.

### 2.2. Performance Requirements of Sensors Simulating Capabilities of the Human Sense

Pressure and tactile sensors for robotic and minimally invasive surgery must cover the typical surgical force range with sufficient resolution, speed, and accuracy to detect clinically relevant changes in tissue load without saturating or missing peaks. Typical medical interaction forces in MIS fall within about 0–25 N but may reach 40 N during certain laparoscopic tasks. Accordingly, sensors are generally designed to accommodate force ranges of approximately ±10–40 N, depending on the instrument and procedure [5,21].

Literature reviews recommend a minimum force sensitivity of approximately 0.2 N for practical force feedback, whereas state-of-the-art micro-electromechanical system (MEMS) and optical tactile sensors can achieve sub-millinewton resolution (<1 mN) across multiple axes, exceeding human perception and enabling fine tissue differentiation [22]. Response time and bandwidth must support real-time control and haptic rendering; typical design targets include response times on the order of 1 ms and bandwidth of at least several hertz for standard grasping tasks, with higher bandwidths desirable for rapid tool motions or vibrotactile feedback. Sensors also must maintain accuracy under dynamic loading conditions, distinguishing static from dynamic forces as well as normal and shear components during palpation, sliding, or traction manoeuvres.

Key performance indicators include linearity, repeatability, and minimal cross-talk between axes, as errors in multi-axis force vectors can compromise both haptic feedback fidelity and tissue property estimation algorithms.

Although different surgical procedures may require tailored sensor configurations or adjustable gain settings, all systems must meet stringent constraints related to miniaturisation (typically 5–8 mm instrument diameter), sterilisability, biocompatibility, and mechanical robustness in addition to quantitative performance requirements [5,20].

### 2.3. Current Issues with RAS Systems

**Figure 1:**
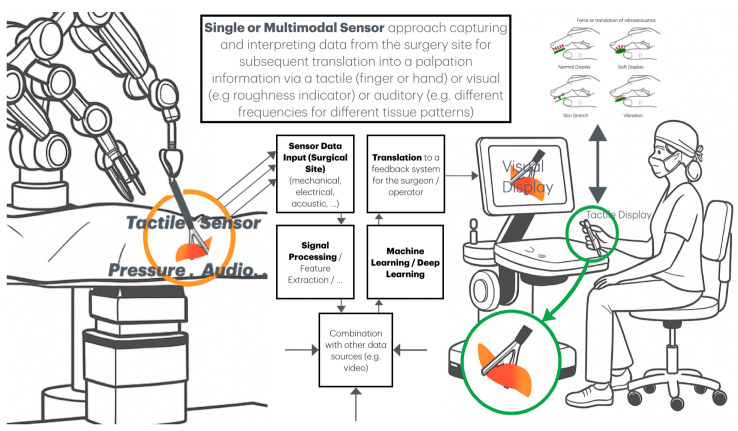
Show a general setup of a robotoc surgery system. Sensors are integrated in the surgical tool or attached to the shaft (e.g., force, pressure, audio, capacitive, or others), obtain information about the tissue and translate it via a tactile display (or as auditory or visual combination) to the guiding hand or finger of the tool-controlling surgeon. Other data sources may be combined and machine learning algorithms used to translate the information to a tactile sensation (combined with visual and audio cues).

Despite substantial advances in robotic-assisted surgery, several challenges continue to limit widespread adoption and scalability (see Table 4 and Figure 1). Current systems are expensive, large, and technically complex and are restricted to specific procedures or clinical domains. However, the most significant barrier to wider implementation remains the extensive training required for human operators. Consequently, the pool of fully trained robotic surgeons is small and concentrated in selected centres primarily located in the healthcare systems of high-income nations.

A key reason for the prolonged training is the continued lack of tactile feedback, as surgeons rely solely on visual input from a camera with a limited field of view, only occasionally supplemented by on-site diagnostic imaging. Without direct sensory feedback, the cognitive load on the operator remains high, thereby extending training times and limiting scalability.

***“Integrating essential tissue information—either for human interpretation or as sensor input for future semi-autonomous or autonomous systems—could reduce training demands and decrease human dependence.”***

This shift would likely encourage wider system adoption. As more RAS units are deployed, increased competition may drive down costs, spurring further innovation. Although this economic argument may seem simplistic, the trajectory of RAS innovation depends heavily on advancing automation and reducing reliance on human expertise.

Another significant economic barrier is the lengthy setup and preparation time required for RAS, which often exceeds that of conventional minimally invasive surgery (MIS). Furthermore, current evidence does not demonstrate that RAS is broadly superior to laparoscopic surgery in terms of clinical outcomes [23,24]. While robotic systems offer clear benefits in specific procedures—particularly in urology and gynaecology [25]—the advantages for many surgeries are marginal and often do not justify the substantially higher costs (see Table 4, last column).

Although randomised trials have yet to confirm an overarching clinical advantage of RAS, emerging data suggest improved outcomes in select procedures. To support further adoption, rigorous, procedure-specific studies and cost-effectiveness analyses are essential. Addressing the absence of tactile feedback—initially for human operators and eventually for autonomous systems—could enhance surgical accuracy, safety, and outcomes.

The following section explores promising research directions, focusing on emerging sensor technologies and the integration of artificial intelligence and deep learning models.

### 2.4. Importance of Palpation Information and Tissue Feedback (Haptics)

Recent advances in robotic surgery and bioengineering aim to enhance the intuitive and immersive experience for surgeons, enabling more precise manipulation and ultimately improving clinical outcomes and patient safety [14,26].

Several research efforts are underway to address this gap (see Table 5 for details). However, most of these approaches remain in early development stages, lack regulatory approval, and often introduce added cost, system complexity, and training requirements.

Many solutions are only effective when integrated and combined with complementary technologies—for example, force sensors (see item 1 in Table 5), advanced surgical instruments with embedded sensors (item 2), and artificial intelligence or deep learning algorithms (item 5) [26].

An additional, yet underrepresented, area is the integration of external diagnostic imaging systems. These can expand the limited field of view provided by RAS cameras, which typically focus only on the immediate surgical site and tool interactions. Imaging modalities such as X-ray, MRI, and ultrasound offer valuable support for localisation and guidance but also introduce additional costs and workflow complexity.

Importantly, subtle tissue abnormalities—such as micro-tears in ligaments, tendons, or fascia—may elude imaging detection but can be identified through palpation, which provides critical diagnostic information aligned with a patient’s symptoms and physical findings [27,28].

Palpation-related sensing is especially critical in tasks where tactile cues strongly influence intra-operative decisions but vision alone is insufficient. An example is safe tissue grasping and traction of the bowel, vessels, or mesentery without crushing or tearing as well as maintaining traction during dissection. In these contexts, jaw-integrated normal and shear force sensors or high-rate force sensing at the instrument wrist could be applied. This information could be translated into a real-time force reflection or vibrotactile warnings when approaching unsafe force thresholds or visual force bars or traffic-light overlays [28]. Such palpation sensing technologies could reduce peak forces and tissue injury and enable safer manipulation of friable tissues.

Another example is the vessel and duct identification and handling to avoid inadvertent avulsion or clip misplacement. This could be addressed by high-sensitivity tactile/pressure arrays capable of assessing diameter and wall stiffness with optional temperature sensing. These inputs could generate pulsation-modulated haptic or visual cues indicating vessel presence and approximate calibre including alerts when traction exceeds safe levels. With appropriate palpation sensing, it would be possible to distinguish and recognise vessels and ducts, perform safer clipping, and perform autonomous safety checks (e.g., “do not cut if pulsatile structure detected”).

Additional applications where palpation sensing could provide benefits are verification of remaining within the correct anatomical plane (e.g., between fascia layers or around a tumour capsule) during blunt dissection and palpation-guided needle placement and biopsy [29].

***“Palpation sensation can provide information that may not even be visible on imaging, such as subtle tenderness or small soft tissue abnormalities.”***

**Table 5:** Haptic and palpation solution ideas and technologies that are currently being researched.

Haptic Feedback + Palpation Sensation Techn.	Details	Drawback/Comment
**Force—Sensor Integrations** [3,4,5,6]	Integrating miniaturised force and tactile sensors onto surgical instruments to measure real-time tissue resistance and interaction forces. Multimodal Haptics: Combine vibrotactile, kinesthetic, and thermal feedback to simulate realistic tissue textures and resistance.	Dedicated tools needed Expensive to implement May limit tool functionality Additional cables/electronics
**Sensorized Surgical Instruments** [14,15]	MEMS and Fiber-optic Sensors: Utilising micro-electromechanical systems (MEMS) or optical fibre-based force sensors for precise and reliable measurement without increasing instrument size significantly.	See above but less expensive and with lower negative effect on functionality.
**Wearable and Tactile Interfaces** [21,24,26]	Glove-based Feedback Devices: Developing wearable gloves or fingertip sensors that translate measured tissue interactions into tactile sensations directly delivered to surgeons. Exoskeleton-based Systems: Employing exoskeleton devices providing force-feedback and natural motion sensations directly to the surgeon’s hands or arms.	Expensive to produce and maintain Adds complexity Requires additional training
**Virtual Reality (VR) and Augmented Reality (AR) Integration** [3,4,5,6]	Virtual Haptic Rendering: Integrating VR environments with computational algorithms that simulate tissue interactions to offer realistic haptic sensations virtually. AR-enhanced Force Feedback: Overlaying force interaction information directly onto surgical video streams, assisting surgeons visually when physical haptic sensations are unavailable.	Simulation is always worse than actual data, but the combination with advanced sensor information and artificial intelligence and deep learning is promising. Likely expensive and complex.
**Artificial Intelligence (AI) and Deep Learning (DL)** [30,31,32]	Leveraging machine learning algorithms to predict tissue stiffness and force interactions based on visual data, motion tracking, and instrument movements. Deep Learning-based Tactile Feedback Generation: Training neural networks to accurately infer and reproduce realistic tactile sensations from visual and robotic data.	Requires data input from the actual surgery (sensors, imaging). Very promising but still in research modus.
**Soft Robotics** [31,32,33,34]	Flexible Instrumentation: Development of soft or compliant robotic instruments capable of passively adapting and providing intrinsic haptic cues based on deformation when contacting tissues. Bio-inspired Robotics: Designing instruments inspired by biological structures (e.g., tactile hairs, mechanoreceptors) to naturally sense and transmit haptic feedback.	This is still research. No system close to actual clinical use. Unclear on how long it will take before actual commercial availability.
**Remote Tele-operation Improvements**	Low-latency Communication Systems: Ensuring near-instantaneous feedback loops using improved communication protocols to preserve realistic haptic sensations, especially critical in remote surgeries.	No drawback. New 6G mobile networks promise to have little to no latency.
**Hybrid Feedback Approaches** [4,5,6,14]	Visual–Haptic Fusion: Creating hybrid sensory modalities combining visual cues, audio feedback, and tactile signals to reinforce surgeons’ spatial awareness and precision.	More and more complex but will be the basis for more autonomy. Initially expensive with the hope to become cheaper with volume production.

## 3. Sensor Technologies and Solutions for Palpation Sensing

A variety of sensor technologies can be integrated into robotic surgical instruments to restore or approximate tactile and palpation feedback. Each approach has distinct advantages, limitations, and levels of technological maturity. This section reviews the principal categories of palpation-related sensor technologies and their current applications.

### 3.1. Simulators

Current approaches to compensate for the absence of haptic or palpation sensors in surgical tools primarily rely on extensive training with laparoscopic and robotic-assisted surgery (RAS) simulators. These systems train surgeons to cognitively interpret visual cues—such as tissue deformation in the video feed—as a surrogate for tactile feedback.

Despite the lack of true haptic feedback in most RAS platforms, laparoscopic and arthroscopic simulators and tools still provide a limited degree of tactile coupling between the surgeon and the operative site. When combined with visual input, this residual feedback is often sufficient to ensure surgical safety and precision. This situation changes fundamentally in RAS systems, where the surgical tool is physically decoupled from the surgical operator (see Figure 1).

As RAS systems do not provide direct haptic feedback, visual interpretation remains the primary method for assessing surface characteristics. Surgical simulators replicate real surgical instruments within artificial environments composed of synthetic organs, tendons, muscles, and tissues. However, most simulators lack vascular structures and, therefore, do not replicate perfusion-related and other dynamic physiological effects.

***“Laparoscopic simulators have become valuable tools in medical education and preoperative planning, incorporating advanced bioengineering to improve training outcomes.”***

A major area of development for these simulators has been the integration of haptic feedback systems to enhance the realism of the simulated tissue interactions.

Emerging bio-inspired palpation systems combine multiple feedback modalities and have been shown to help novice surgeons reduce applied grip force during training, indicating improved sensory awareness. In cases where haptic feedback is not feasible, sensory substitution techniques—such as visual overlays or auditory cues—have been explored to convey force-related information. However, studies suggest these substitutes are often perceived as unnatural and appear less effective at conveying information about subsurface tissue structures.

Research further indicates that, while haptic feedback is critical for force-intensive tasks, it plays a lesser role in orientation-driven procedures like suturing and knot tying, which primarily rely on precision gestures and visual guidance [30]. This finding highlights the continued need to improve alternative sensory feedback strategies.

### 3.2. Sensor Technologies and Feedback Concepts

The sensor technologies described below are increasingly being integrated into minimally invasive surgical instruments and closely resemble those used in contemporary RAS platforms. Their adoption is expected to become standard in both domains. Sensors are typically integrated at the distal tip of the surgical instrument and, in some cases, along the shaft. Acquired data is then relayed to the surgeon through various feedback modalities, including vibration, skin stretch on the fingertip, or auditory signals via tactile displays (see Figure 1).

### 3.3. Force and Tactile Sensors

Force sensors measure the magnitude and direction of forces applied between the surgical instrument and tissue. They can be integrated at various locations: the distal instrument tip, along the instrument shaft, at the proximal end of the instrument outside the patient, or at the master manipulator console. Distal force sensing provides the most direct measurement of tissue interaction forces and is, therefore, particularly relevant for surgeon feedback and autonomous control.

Tactile sensors, in contrast, measure spatially distributed pressure or contact information across a surface area, mimicking the density of mechanoreceptors in human skin [31,32].

Modern tactile sensors employ various technologies like:

- MEMS (Micro-electromechanical Systems) pressure sensors with integrated electronics;
- Optical tactile sensors using light deflection or capacitance changes;
- Strain gauge arrays for distributed pressure mapping;
- Capacitive sensors for contact detection and soft-touch applications;
- Resistive sensors for cost-effective, simple implementations.

A key advantage of force and tactile sensors is their ability to provide quantitative data suitable for direct integration with artificial intelligence algorithms for tissue classification and autonomous decision-making. Key challenges include miniaturisation for integration into small-diameter instruments, ensuring biocompatibility and sterilisability, and managing signal noise and drift during prolonged use. Cable routing and connector design are also critical considerations, as sensors must be protected from the surgical environment while preserving signal integrity [31].

### 3.4. Vibroacoustic and Audio Sensing

A novel approach involves placing audio sensors on the instrument shaft (proximal) to capture vibroacoustic signals during tool–tissue interactions. These low-cost sensors have shown potential in detecting tissue stiffness, surface texture, and even underlying perfusion-related sounds [6]. Vibroacoustic data contain rich information about tissue properties, including stiffness, density, and internal structures.

**Figure 2:**
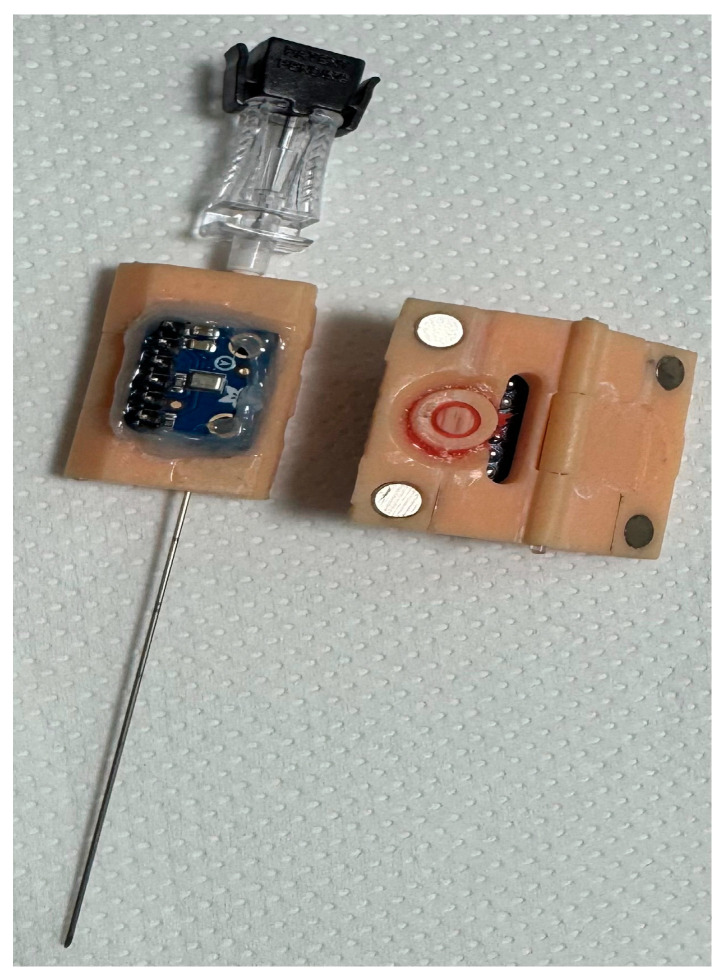
It shows a MEMS—micro-electromechanical system—audio sensor based on the principle as shown in Figure 3G,J. This prototype was attached via magnets to the proximal end of a commercial aspiration needle.

Audio-based approaches typically use high-frequency transducers or accelerometers mounted on surgical instruments to detect tissue-induced vibration patterns. Advantages include non-contact assessment of remote or subsurface tissue properties (e.g., subsurface lesions), minimal added sensor mass or footprint, and compatibility with existing surgical instruments through retrofitting. Figure 2 shows a prototype audio sensor that is attached with magnets around an aspiration needle [6].

However, several technical challenges remain, including shielding of acoustic noise from surgical environments, variability in tissue acoustic properties, and the need for advanced signal processing techniques to extract clinically meaningful features. Current research is focused on improving signal-to-noise ratios and validating the diagnostic relevance of specific vibration signatures for tissue differentiation.

### 3.5. Other Sensing Approaches

**Optical sensors**, including fibre Bragg grating (FBG) sensors and other light-based approaches, can be miniaturised to very small diameters and integrated directly into hollow needles or endoscopes. These sensors measure strain, pressure, or wavelength shifts induced by tissue interaction. They can be easily miniaturised, are immune to electromagnetic interference, have multiplexing capabilities and can be directly integrated into hollow surgical instruments.

Additional sensing approaches are:

**Active Force Feedback Systems:** Platforms such as the da Vinci 5 now provide real-time force feedback, enabling surgeons to perceive pressure, tension, and resistance during tasks such as dissection, retraction, and suturing. Studies show this reduces applied tissue force by up to 43%, enhancing both safety and accuracy [31,32].

**Combined Kinesthetic and Tactile Feedback:** Modern training systems integrate both force (kinesthetic) and texture/grip (tactile) feedback, offering a more immersive and realistic environment for skill acquisition and practice [33].

**Multimodal Feedback Interfaces:** Some platforms combine haptic, visual, and auditory cues to create richer and more intuitive user interfaces that improve situational awareness and procedural control [31,32].

**AI-Augmented Haptics:** Machine learning algorithms are increasingly applied to large volumes of tactile and force data, enabling systems to recommend optimal force levels, flag potential risks, or even partially automate specific manoeuvres. This augmentation supports more informed, adaptive, and consistent surgical performance [34].

Table 6 summarises key technologies likely to contribute to palpation sensing and tactile feedback in robotic-assisted surgical (RAS) systems.

**Figure 3:**
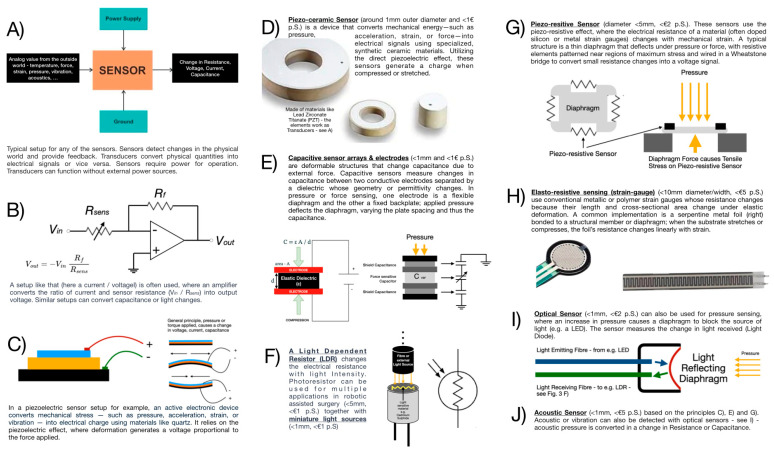
An incomplete selection of sensors that are applicable for the needed functions associated with palpation in RAS as well as some basic functional principles. (**A**) General principle of a sensor acquiring analogue real-world data and converting it into a resistance change, voltage, current, and capacitance to be read by the sensor electronics. (**B**) General principle of converting the change (in this case a resistive change) that leads to a change in the output voltage. (**C**) Shown is the principle of a piezoelectric sensor that converts mechanical stress (pressure, friction, shear) in a changed electrical charge. (**D**,**E**) The working principle and schemata of piezoceramic and capacitive sensors. (**F**) The light-dependent resistor principle is shown that changes the electrical resistance based on light intensity (can also be used in combination with (**I**)). (**G**) Piezoresistive sensors changing their electrical resistance and (**H**) elastoresistive ones that change electrical resistance due to mechanical influence. (**I**) The principle of an optical sensor for pressure measurement and (**J**) the schemata of an acoustic sensor based on the piezoelectric principle (see (**C**)). Indications of current minimum sensor size and cost per sensor are also included.

Figure 3 illustrates representative sensors, their underlying operating principle and indicative size and cost ranges.

### 3.6. Dedicated Robotic Palpation Devices

Recent advancements have focused on robotic tissue palpation devices that rapidly and non-invasively quantify the stiffness of soft tissues. Designed as intra-operative tools, these devices assist surgeons in localising and assessing pathological tissues, such as tumours, during robot-assisted minimally invasive surgery (RMIS) [3].

By providing tactile feedback, these systems enhance real-time decision-making and address the longstanding limitation of inadequate haptic feedback in robotic platforms [20]. One notable example features a deployable stiffness-sensing probe mounted on a custom wire-driven steerable catheter. This probe integrates a thin-film force sensor and specialised contact electrodes, enabling accurate tissue stiffness measurements. Its design supports multi-directional movements, including linear displacement and rotation, to effectively access the target tissue surface and enable controlled palpation trajectories.

A significant innovation in this domain is the development of wireless palpation devices using magnetic coupling for communication. These systems allow intra-operative palpation without extensive incisions, making them ideal for minimally invasive procedures. Typically comprising an external positioner and a disposable internal palpation device, the system transmits real-time data on force and tissue compression as the components interact remotely [14,35]. This approach enhances patient safety and comfort while enabling the detection of abnormalities, such as tumour growth, during surgery and supports repeated intra-operative assessments without additional tissue trauma.

Integrating advanced haptic feedback systems is expected to significantly enhance palpation capabilities. Efforts to create more intuitive and immersive tools aim to improve surgical outcomes and bolster surgeon confidence in performing complex manoeuvres [36,37].

### 3.7. Integration of Machine Learning and Imaging Technologies

The incorporation of machine learning algorithms into robotic platforms further improves surgical outcomes by delivering real-time analytics and guidance. Surgeons can leverage augmented reality (AR) and virtual reality (VR) to access high-resolution, real-time imaging of the surgical site, enhancing navigation and precision. Combined with 3D imaging, these technologies offer superior visualisation of anatomical structures and assist in characterising tissue properties during minimally invasive procedures.

While large language models (LLMs) and related foundation models currently focus mainly on perception, planning, and workflow support, force/torque and other surgical sensors are typically corrected using dedicated calibration or deep learning regression models that take raw voltages/loads and estimate drift and disturbance [38,39]. Foundation models for robotic surgery (vision–language–action transformers, surgical scene understanding models, and depth-estimation adapters) are being developed to generalise across different anatomies, tools, and workflows, effectively learning invariances or adaptation strategies to inter-patient and inter-case variability [39].

Although LLMs in surgery and medicine are increasingly applied to decision support, report generation, education, and higher-level autonomy orchestration, they are not used in validated clinical settings for remapping tactile signals to account for individual surgeon perception differences.

### 3.8. Multimodal Integration of Advanced Sensing Technologies

The most promising approaches combine multiple sensing modalities with intra-operative imaging and augmented reality visualisation. For example, a robotic system might combine and integrate:

- Force and tactile sensors at the instrument tip;
- Vibroacoustic sensing for tissue characterisation;
- Intra-operative ultrasound or other imaging for spatial context;
- Machine learning models trained on multimodal data;
- An augmented reality overlay displaying predicted tissue properties or danger zones, with haptic feedback delivered via console-mounted haptic interfaces or vibrotactile wearables.

Such integrated systems can provide surgeons with a much richer understanding of the surgical site and tissue properties, akin to the information available in open surgery. Research into optimal sensor fusion, real-time processing, and intuitive user interfaces is ongoing.

### 3.9. Sensor Limitations

Perhaps the most significant hurdle is the integration and miniaturisation of sensors. The surgical environment imposes strict constraints on sensor size, shape, cost, biocompatibility, and sterilisability, making it difficult to incorporate force sensors into existing robotic instruments. Retrofitting sensors onto tools not originally designed for them is particularly challenging, often necessitating complex and expensive custom designs [11].

Sensor-specific limitations also persist. For instance:

**Piezoelectric sensors** are highly responsive to dynamic forces but are less effective at detecting static forces. **Capacitive sensors** are sensitive to environmental conditions such as humidity and temperature. **Optical sensors**, while immune to electrical interference and very precise, are less common due to implementation challenges in surgical settings. **Audio sensors** are easy to integrate and cost-effective, but they require new interfaces to translate acoustic signals into actionable feedback for the surgeon.

In addition to these technical issues, environmental and human variability must be considered. Differences in tissue properties across patients—and even within a single patient—complicate force data interpretation. Human factors such as hand strength, technique, and fatigue further contribute to inconsistencies. Moreover, dynamic forces generated by robotic systems can mask the subtle forces involved in tissue interaction, limiting the fidelity of force estimation [11].

To mitigate these limitations, compensation techniques such as calibration, signal filtering (e.g., low-pass, Kalman, or adaptive filters), and advanced algorithms are employed. However, none of these methods offer a fully reliable solution [30,40].

Finally, a universal standard for evaluating force and sensor performance in robotic surgery is urgently needed. Validation of sensing technologies across diverse clinical settings is essential to ensure safety, consistency, and effectiveness [13,14].

Table 7 summarises the key limitations of sensors.

**Figure 4:**
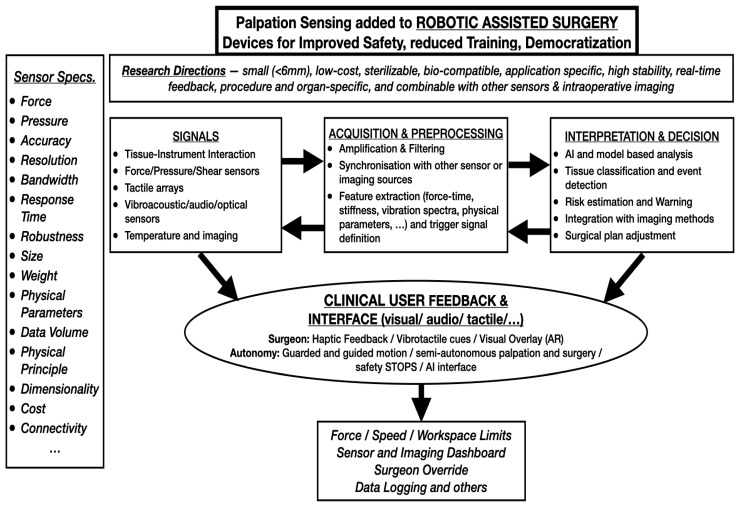
A summary figure showing the different sensor specifications that need to be considered and evaluated based on the clinical need and integration requirements. The sensor signals will require a preprocessing step after acquisition and a subsequent data interpretation step that provides risk estimations and warnings as well as vibrotactile cues and is combined with other sensor and imaging data.

Figure 4, based on the underlying research and development hypothesis, illustrates the specifications that must be evaluated according to clinical application, signal type, and preprocessing requirements, leading—via AI-based evaluation and processing—to different forms of interpretation and decision support that are communicated to the clinical user and contribute to increasing levels of RAS autonomy.

### 3.10. How Close Are Current Technologies to Replicating Human Tactile Sensation in Surgery?

Current robotic-assisted surgical systems have made notable strides in restoring aspects of tactile sensation, yet they still fall short of replicating the richness and nuance of human touch experienced in open surgery.

Recent advancements provide surgeons with real-time data on grasping forces, tissue stiffness, and thickness. For instance, innovative “off-the-jaw” sensing systems now deliver objective tactile feedback, enhancing precision and safety in minimally invasive procedures [11].

Some state-of-the-art surgical and training systems integrate both kinesthetic (force) and tactile (surface and grip) feedback, achieving up to 95% accuracy in force/torque detection. This improves simulation fidelity and surgical control [42]. Additionally, compact, lightweight, wireless wearable devices have been developed to reproduce a range of tactile sensations—vibration, pressure, stretching, and twisting—enabling programmable, nuanced feedback [30].

Incorporating tactile feedback in robotic systems has been shown to reduce excessive grasping forces and minimise tissue damage, particularly aiding novice surgeons in mastering delicate manoeuvres more quickly [24,43,44].

Nonetheless, significant limitations remain. Human tactile perception involves an intricate integration of pressure, vibration, texture, temperature, and proprioception. Most current systems provide only limited force and basic tactile cues, lacking the full sensory spectrum of a surgeon’s hands [30,40,45]. Furthermore, many advanced haptic systems are challenging to incorporate into surgical tools due to constraints related to size, sterilisation, and biocompatibility [46].

While some technologies approach the precision of human touch, achieving a truly “transparent” experience—where the surgeon feels as though they are directly manipulating tissue—remains a technical hurdle. However, this limitation may diminish with the rise in systems incorporating higher levels of autonomy.

***“With many different approaches and growing competition there is also the need to develop and adopt a universal standard for haptic feedback in surgical robotics, and widespread clinical use of high-fidelity tactile feedback is still emerging.”***

## 4. Conclusions

The integration of palpation sense and haptic feedback into robotic-assisted surgery (RAS) systems is a critical step toward enhancing surgical safety, precision, and outcomes [11,43]. Current RAS platforms rely heavily on visual input, limiting their capabilities and maintaining a high dependence on highly trained human operators. This reliance hinders scalability and drives up procedural costs. The lack of tactile information not only impairs tissue characterisation but also increases the risk of inadvertent tissue damage [30,46].

Although significant progress has been made in force sensors, tactile feedback systems, and AI-driven decision-support tools, challenges such as sensor miniaturisation, integration, biocompatibility, and real-time data processing remain. Emerging solutions—such as proximally attached audio sensors and robotic palpation tools—show promise, particularly when paired with AI for real-time interpretation and decision-making [11,13,40].

Enabling effective palpation and haptic feedback is essential for advancing toward semi-autonomous and fully autonomous surgical platforms. Such advancements would improve procedural accuracy and safety, reduce surgeon workload, shorten training times, and expand access to high-quality surgical care globally. To achieve this, continued interdisciplinary research, standardisation efforts, and cost-conscious engineering are vital [44].

Numerous promising sensor approaches in research settings, though not discussed in detail here, are expected to yield valuable multimodal data to support the simulation of palpation in the near future [45,47,48,49,50,51,52,53,54,55,56,57,58].

For further development in minimally invasive surgery (MIS) and RAS, key priorities include cost-effectiveness and compact design. Without these, haptic feedback systems are unlikely to achieve widespread adoption in training or clinical use [33]. Moreover, many sensors come into contact with patients and must, therefore, be not only small and robust but also biocompatible and capable of withstanding sterilisation processes [59].

As these technologies will integrate to more sophisticated haptic feedback and collaborative functionalities, a novel definition of a RAS system could be:

***“A surgical robot is an intelligent, learning-enabled system capable of autonomously or collaboratively performing or assisting surgical procedures, using real-time data, adaptive control, and integrated sensing to optimise precision, safety, and patient outcomes.”***

### 4.1. Development Roadmap Suggestion and Effect Prediction

A practical roadmap toward palpation-enabled, more autonomous RAS can be structured around a few prioritised research directions that directly follow from this manuscript’s arguments.

These include procedure- and organ-specific requirements for force/pressure range, resolution, bandwidth, accuracy, robustness, and sterilisation for palpation sensors, derived from real surgical data rather than benchtop assumptions. Standardised test phantoms and benchmarking protocols for tissue “feel” (force–displacement, stiffness, viscoelasticity) should map sensor outputs to surgeon psychophysical scales and task performance.

Miniaturisation remains a critical issue. Compact, biocompatible, sterilisable force/tactile/vibroacoustic sensors that fit into 5–8 mm inner diameter instruments or detachable palpation probes, with low drift and stable calibration over repeated sterilisation cycles, are needed. Initially, for the feasibility tests, that is obviously not a mandatory requirement, but if it is foreseeable that the size requirements cannot be met in the near future, than it likely would make more sense to look for early alternatives. Modular sensorised tools and wireless or magnetically coupled palpation devices may offer practical interim solutions that avoid major platform redesign. AI-based multimodal sensor data will be essential, with algorithms that fuse palpation data (force, compliance, vibration spectra) with endoscopic video and external/intra-operative imaging (US, CT, MRI, X-ray) for improved tissue classification, lesion localisation, and margin assessment.

The AI models must be trained on synchronised video–palpation–outcome datasets to predict pathology, depth of inclusions, and safe force envelopes and to provide real-time decision support during MIS/RAS. Equally important is the human–machine and personalisation interface. How will the data be communicated or transferred to the human user? Will tissue surface information be translated into a tactile sensation or rather into a video or audio interface? And in that context, it will be necessary to implement and validate individualised calibration techniques, where sensor outputs are mapped to each surgeon’s just-noticeable differences and preferred feedback gains, leveraging the fact that sensor sensitivity exceeds human thresholds.

Ultimately, these advances will enable higher RAS autonomy and safety, supporting semi-autonomous tasks such as autonomous palpation scans, guarded dissection, and safe retraction under human supervision.

Integration with digital twins and predictive tissue models will further support simulation, rehearsal, and real-time risk estimation, forming a basis for progression along surgical autonomy scales, moving the LASR towards level 3 and beyond. In parallel to the development and translation efforts, regulatory-ready performance metrics need to be developed along with reporting standards for palpation/haptic subsystems in RAS, including safety, reliability, failure modes, and interoperability with existing platforms.

### 4.2. Future Directions and Technical Translation Priorities

Future research in robotic palpation and haptic sensing in RAS should follow a clear roadmap that tackles three linked priorities: sterilisation-ready hardware, multimodal AI-augmented feedback, and clinically validated platforms that support higher autonomy. On the hardware side, the immediate bottleneck is the development of compact, biocompatible, sterilisable force–tactile–vibroacoustic sensors that fit 5–8 mm instruments or detachable palpation probes while maintaining low drift and stable calibration over repeated cleaning cycles.

Promising strategies include robust encapsulation with established medical-grade materials, hermetically sealed optical or MEMS packages, and proximally mounted vibroacoustic sensors or magnetically coupled palpation probes that reduce direct tissue contact and simplify sterilisation. In parallel, modular sensorised tools and standardised performance benchmarks, covering force–displacement behaviour, bandwidth, cross-talk, and post-sterilisation stability, are needed to integrate palpation capabilities into existing robots and to meet regulatory expectations.

Finally, synchronised video–palpation–outcome datasets should be used to train AI models that fuse force, vibration, and imaging data, enabling semi-autonomous behaviours such as guarded dissection and autonomous palpation scans, which can then be evaluated in multi-centre trials and health-economic studies to demonstrate reductions in tissue injury, training time, and episode-of-care costs.

## Figures and Tables

**Table 1:** Palpation and loss-of-touch-related limitations and impact on surgery [1,2,3,4,5,6,7,8].

Limitation	Impact on Surgery
***Lack of haptic feedback [3,4]***	Reduced ability to distinguish tissue types; higher risk of tissue injury
***Limited tissue characterisation [1,2]***	Harder to identify pathology and subtle tissue changes
***Increased risk of tissue damage [8]***	Excessive force may be applied unknowingly
***Technical/practical barriers [7]***	Slow adoption of advanced feedback systems

**Table 2:** Levels of autonomy in surgical robotics [13].

Level	Description	Current Clinical Use
0	No Autonomy—Manual tools, no robotic assistance	Not robotic, typical minimal invasive surgery approach
1	Robot Assistance—Surgeon in full control, robot provides physical/cognitive aid	Most common
2	Task Autonomy—Robot can autonomously perform specific tasks when instructed	Still rare but emerging
3	Conditional Autonomy—Robot can plan tasks, adjust actions with surgeon oversight	Very limited, advanced systems only and very limited applications
4	High Autonomy—Robot plans and executes sequences with minimal human input	Not available and in clinical use yet
5	Full Autonomy—Robot performs entire procedures independently	Not available and in clinical use yet

**Table 3:** Aspects of touching and technologies implemented in RAS systems [3,4,5,6,17,18,19,20].

Aspect of Touch	Human Sensation	Current Robotic Technologies	Status
***Pressure [3,5]***	Highly nuanced	Basic force feedback; some tactile sensors	Partial replication
***Vibration [6]***	Wide frequency range	Limited; some wearable devices	Emerging
***Texture [4,6]***	Fine discrimination	Not widely replicated	Limited
***Temperature [5,18,20]***	Higher or lower than normal	Not replicated	Absent
***Proprioception [4,17]***	Natural, continuous	Kinesthetic feedback via sensors	Partial
***Multi-directional Feedback [4]***	Yes	Some wearable devices; limited in tools	Emerging

**Table 4:** The different issues with robotic-assisted surgery (RAS) and its systems at the moment and as compared to more conventional minimal invasive surgery (MIS) approaches (extracted from [21,22,23,24,25]).

RAS Issues		Compared to More Conventional MIS
***High Cost and Economic Barriers***	High initial investment High operational expenses Limited availability in low-resource settings	Much lower investment and operational cost and widely available Tools reusable after sterilisation Widely available globally
***Lack of Haptic Feedback***	Surgeons rely on visual cues at the moment Force feedback sensors are offered in the newest generation only Increased risk of inadvertent damage	There is a direct connection—through the tool—between the surgeon and the tissue Training is relatively straight forward using simulators
***Complex Training and Learning Curve***	Extensive training required for proficiency High dropout rates among trainees Limited availability of training programs	All medical students receive minimal invasive surgery training Can easily be updated using simulators
***Limited Versatility and Adaptability***	Often, systems are optimised for specific procedures Difficult to adapt to changing situations during the surgery	Systems and tools are much easier; adaptable and dedicated surgical tools are widely available
***Technical and Mechanical Constraints***	Large physical footprint with access restrictions Limited degree of freedom may restrict dexterity and restrict movement	Requires relatively little space (endoscopic tower) in a surgery room and can be moved relatively easily
***Dependence on Human Operator***	Systems are primarily tele-operated No autonomous system operation—heavily relies on the human operator	Surgeon’s ability and experience also very important, but there are typically several experts available
***Latency and Responsiveness***	Small but perceptible delay between operator input and tool movement Worse for remote telesurgeries	No latency No remote operation possible
***Safety Concerns***	Risk of mechanical failure, software issues or instrument failure Emergency protocols needed	Instrument failure can also happen, but typically, replacement is available immediately
***Limited Integration and Interoperability***	Difficulty integrating with other equipment Imaging modalities Health IT systems	Only the endoscopic tower has to be integrated. Standard connection protocols (e.g., DICOM for image files)
***Regulatory and Liability Challenges***	Complex regulatory approvals slow innovation No real clarity on liability in case of malfunction	Innovations also need to be approved with similar timelines

**Table 6:** Key technologies for palpation.

Technology Area	Example/Functionality/Advantage
***Force/Tactile Sensors [31]***	Real-time measurement of push, pull, grip, and texture
***Active Force Feedback [31,32]***	Surgeons feel resistance/tension during tasks
***Multimodal Feedback [14,31,32]***	Combines haptic, visual, and auditory cues
***AI-Augmented Haptics [34]***	Machine learning interprets and guides tactile feedback
***Vibroacoustic Sensing [6]***	Can be mounted proximal, low-cost, rich sensor data

**Table 7:** Key limitations of sensors for palpation and haptic feedback [2,13,41].

Limitation	Description
***Sensor noise and drift***	Electrical/mechanical noise and drift affect measurement accuracy
***Mechanical compliance***	Robot and tissue deformation introduce uncertainty
***Integration challenges***	Size, biocompatibility, and sterilisability constraints in surgical tools
***Sensor-specific weaknesses***	Each sensor type has unique limitations
***Tissue and human variability***	Nonlinear, patient-specific tissue properties and human factors complicate calibration
***Dynamic force masking***	Robotic system dynamics can obscure subtle tissue interaction forces
***Incomplete compensation***	Calibration and filtering help but do not fully resolve uncertainties
***Lack of standardisation***	No universal benchmarks or widespread clinical validation

## Data Availability

No new data were created or analyzed in this study.
